# Poly-d,l-lactic Acid (PDLLA) Application in Dermatology: A Literature Review

**DOI:** 10.3390/polym16182583

**Published:** 2024-09-13

**Authors:** Kar Wai Alvin Lee, Lisa Kwin Wah Chan, Angela Wai Kay Lee, Cheuk Hung Lee, Sky Tin Hau Wong, Kyu-Ho Yi

**Affiliations:** 1 EverKeen Medical Centre, Hong Kong; alvin429@yahoo.com (K.W.A.L.); drchan.everkeen@gmail.com (L.K.W.C.); andylee618@hotmail.com (C.H.L.); 2 The Skin Oracle, Hong Kong; ang3la.l33@gmail.com; 3 Leciel Medical Centre, Hong Kong; drskywong@gmail.com; 4 Division in Anatomy and Developmental Biology, Department of Oral Biology, Human Identification Research Institute, BK21 FOUR Project, Yonsei University College of Dentistry, 50-1 Yonsei-ro, Seodaemun-gu, Seoul 03722, Republic of Korea; 5 Maylin Clinic (Apgujeong), Seoul 06001, Republic of Korea

**Keywords:** poly-d,l-lactic acid, poly lactic acid, filler, biocompatible materials, skin rejuvenation, scar remodeling, dermal fillers

## Abstract

Poly-d,l-lactic acid (PDLLA) is a biodegradable and biocompatible polymer that has garnered significant attention in dermatology due to its unique properties and versatile applications. This literature review offers a comprehensive analysis of PDLLA’s roles in various dermatological conditions and wound-healing applications. PDLLA demonstrates significant benefits in enhancing skin elasticity and firmness, reducing wrinkles, and promoting tissue regeneration and scar remodeling. Its biodegradable properties render it highly suitable for soft tissue augmentation, including facial and breast reconstruction. We discuss the critical importance of understanding PDLLA’s physical and chemical characteristics to optimize its performance and safety, with a focus on how nano- and micro-particulate systems can improve delivery and stability. While potential complications, such as granuloma formation and non-inflammatory nodules, are highlighted, effective monitoring and early intervention strategies are essential. PDLLA’s applications extend beyond dermatology into orthopedics and drug delivery, owing to its superior mechanical stability and biocompatibility. This review underscores the need for ongoing research to fully elucidate the mechanisms of PDLLA and to maximize its therapeutic potential across diverse medical fields.

## 1. Introduction

**Figure 1:**
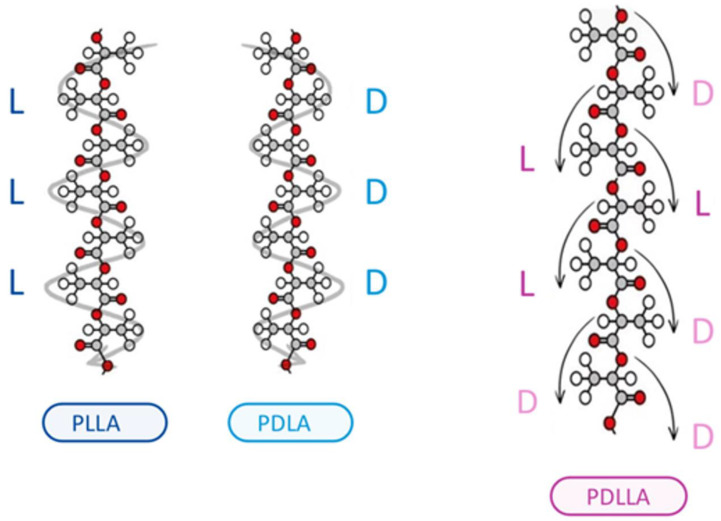
Poly(D,L-lactic acid) (PDLLA) exhibits superior mechanical stability and biocompatibility compared to poly(L-lactic acid) (PLLA) and poly(D-lactic acid) (PDLA) due to its unique stereoisomeric composition, which results in an amorphous polymer structure. PLLA is hemicrystalline and has a regular chain structure, whereas PDLLA is amorphous and has an irregular chain with random distribution of L- and D-lactic acids. This structure prevents the formation of large crystalline regions that are typically found in PDLA and PLLA, making PDLLA less brittle and more resistant to mechanical stress. PDLLA’s homogeneous distribution of D- and L-lactic acid monomers allows for controlled and predictable degradation, which is crucial in biomedical applications where the timing of polymer breakdown affects therapeutic outcomes. Moreover, PDLLA’s gradual and uniform degradation produces smoother degradation products, reducing the likelihood of inflammatory responses, while its primary degradation product, lactic acid, is naturally metabolized by the body, further enhancing its biocompatibility. These properties make PDLLA an advantageous material for use in dermatological treatments and wound-healing applications. The D-form and L-form monomers are bonded in random order, maximizing the contact surface area, which is why PDLLA is widely used in orthopedics and drug delivery.

**Figure 2:**
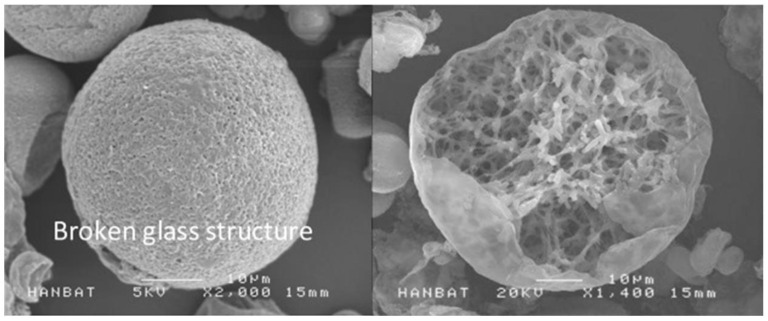
SEM image of the PDLLA (The image was provided in courtesy of VAIM Inc., Republic of Korea with the product Juvelook) [7]. The PDLLA microparticles are spherical in shape with multiple pores on the surface, and have a diameter of 30–70 µm. The microparticle size of both PDLLA monomers make them small enough to pass through an injection needle, but large enough to protect them from phagocytosis. The PLAs may have adverse effect of nodule formation, however, PDLLA produced by VAIM Inc. has foamy structures which can be degrades using energy-based devices.

Poly-d,l-lactic acid (PDLLA) is a biodegradable and biocompatible polymer that has gained significant attention in the field of dermatology in recent years [1]. As a biomaterial, PDLLA has been extensively studied for its potential applications in various medical fields, including dermatology. The unique properties of PDLLA, such as its ability to stimulate collagen synthesis, promote tissue regeneration, and exhibit controlled degradation rates, make it an attractive material for the treatment of various skin-related disorders (Figure 1) [2]. Various polymers are utilized in the medical field to induce collagen production through mild inflammation. However, excessive inflammation can result in complications such as granulomas and nodules. To mitigate these adverse effects, various polymers are being developed. Among them, PDLLA has shown promising features. The outer structure of PDLLA is spherical and foamy, which minimizes damage to surrounding tissue. The inner structure is characterized by a patented reticular and porous design, enhancing biocompatibility and biodegradability. This structure also ensures slow decomposition from the inside, preventing drastic changes in acidity around the particles (Figure 2).

In dermatology, PDLLA has been explored for its potential applications in wound healing, skin rejuvenation, and scar remodeling. Its use in wound healing has been shown to improve wound contraction rates, increase collagen production, and enhance tissue elasticity [3]. Additionally, PDLLA has been used to enhance skin elasticity and reduce fine lines and wrinkles, making it a promising material for skin rejuvenation. Furthermore, its ability to stimulate collagen synthesis and promote tissue regeneration has also been investigated for its potential use in scar remodeling [4].

The literature on PDLLA application in dermatology is rapidly growing, with numerous studies reporting its efficacy and safety in various clinical settings. However, there is a need for a comprehensive review of the current literature to better understand the potential benefits and limitations of PDLLA in dermatology. This literature review aims to provide an overview of the current state of knowledge on PDLLA application in dermatology, including its mechanisms of action, clinical applications, and potential benefits and limitations [5]. By summarizing the existing literature on PDLLA in dermatology, this review aims to provide a comprehensive understanding of the role of PDLLA in the treatment of various skin-related disorders. Keywords including “Poly-d,l-lactic acid”, “Polydioxanone-coated poly(lactic-co-glycolic acid)”, “PDLLA” “Clinical application”, “Clinical implication”, “Clinical use”, “Dermatology”, “Juvelook” were searched in the MEDLINE, PubMed and Ovid databases for relevant studies published on clinical trials, diagnosis and treatment. Some papers were further reviewed using a double-blinding approach, sample size, control usage, randomization usage and objective endpoint measurements. All studies were classified according to the Oxford Center for evidence-based medicine evidence hierarchy [6].

## 2. PDLLA in Aesthetic Application

Poly-D,L-lactic acid (PDLLA) has emerged as a prominent material in the field of aesthetic applications due to its unique properties of biodegradability and biocompatibility. As a polymer, PDLLA stimulates collagen synthesis, which plays a crucial role in skin rejuvenation, scar remodeling, and wound healing. These attributes have made PDLLA a preferred choice for various non-surgical cosmetic treatments aimed at enhancing skin texture, elasticity, and overall appearance. Clinical studies have demonstrated the efficacy of PDLLA in improving skin firmness and hydration, reducing wrinkles and fine lines, and treating conditions such as striae distensae and skin laxity. The versatility of PDLLA extends to its use in combination with other treatments, such as hyaluronic acid and platelet-rich plasma, further enhancing its effectiveness in aesthetic procedures. Moreover, innovative delivery methods, including needle-free injectors and laser-assisted techniques, have been developed to optimize the administration of PDLLA, ensuring minimal invasiveness and patient discomfort. As research continues to advance, PDLLA’s role in aesthetic medicine is likely to expand, offering new possibilities for non-surgical cosmetic enhancements with promising outcomes (Table 1).

Seo et al. [5] investigated the skin rejuvenation effect of a combination of polydioxanone-lactide-co-glycolide (PDLLA, Juvelook, VAIM Inc., Republic of Korea) and non-cross-linked hyaluronic acid (NHA) in human subjects. The combination was applied to the facial skin of 20 participants, and their skin elasticity, firmness, and hydration were evaluated using a series of tests. The results showed that the combination significantly improved skin elasticity and firmness, and increased skin hydration compared to the control group. The study also found that the combination reduced wrinkles and fine lines on the face. The study is a preliminary investigation, and the sample size is small, which limits its generalizability. However, the results suggest that the combination of PDLLA and NHA may be a promising treatment for skin rejuvenation (Level 4).

Ma et al. [8] presented a pilot study on the use of injectable poly-D, L-lactic acid (PDLLA) for rejuvenating the dorsal hand. The authors treated 10 patients with moderate-to-severe dorsal hand skin laxity and wrinkles with PDLLA injections. The treatment was administered at 3-month intervals over a period of 9 months. The results showed significant improvements in skin texture, elasticity, and wrinkle depth, as well as a reduction in skin fold thickness and improvement in skin brightness. The study also evaluated the safety and tolerability of the treatment, finding that it was well tolerated and no serious adverse effects were reported. The authors concluded that PDLLA injections may be a promising treatment for rejuvenating the dorsal hand, with potential applications in other areas of the body as well (Level 2b).

Seo et al. [9] presented a novel approach for treating striae distensae (stretch marks) using laser-induced micro-jet injectors with poly-D, L-lactic acid (PDLLA, Juvelook, VAIM, Republic of Korea). The authors used a combination of ablative fractional CO_2_ laser and PDLLA injections to treat 20 patients with striae distensae on the abdomen. The results showed significant improvements in skin texture, elasticity, and the appearance of stretch marks, with a high patient satisfaction rate. The study also evaluated the safety and tolerability of the treatment, finding that it was well tolerated and no serious adverse effects were reported. The authors proposed that the combination of laser-induced micro-jet injectors and PDLLA injections can improve the delivery of PDLLA into the skin, leading to more effective treatment outcomes. The study suggests that this novel approach may be a promising treatment for striae distensae, offering a more effective and sustainable solution compared to traditional treatments (Level 2b).

Lin et al. [10] presented a pilot study on the use of injectable poly-D, L-lactic acid (PDLLA) for nonsurgical lower eyelid rejuvenation in Asian patients. The authors treated 10 patients with moderate-to-severe lower eyelid skin laxity and wrinkles with PDLLA injections. The treatment was administered at 3-month intervals over a period of 6 months. The results showed significant improvements in skin texture, elasticity, and wrinkle depth, as well as a reduction in skin fold thickness and improvement in skin brightness. The study also evaluated the safety and tolerability of the treatment, finding that it was well tolerated and no serious adverse effects were reported. The authors concluded that PDLLA injections may be a promising treatment for nonsurgical lower eyelid rejuvenation in Asian patients, offering a minimally invasive and relatively painless alternative to surgical procedures (Level 3b).

Li et al. [11] presented a study on the development of biodegradable PLLA/PLGA microspheres/collagen composites for continuous soft tissue augmentation. The authors aimed to create a novel biomaterial that can be used for soft tissue reconstruction, such as breast augmentation or facial reconstruction. The composites were prepared by encapsulating PLLA/PLGA microspheres in a collagen matrix, and their mechanical properties, degradation behavior, and biocompatibility were evaluated. The results showed that the composites exhibited excellent mechanical properties, including high tensile strength and Young’s modulus, and were able to maintain their shape and structure over a period of several months. The authors also demonstrated that the composites were biocompatible and supported cell growth and differentiation. The study suggests that these biodegradable composites could be a promising material for soft tissue augmentation, offering advantages such as controlled degradation and biocompatibility (Level 3a).

Ken et al. [12] presented a study on the long-term morphological evaluation of porous poly-DL-lactic acid (PLA) for soft tissue augmentation. The authors investigated the use of porous PLA as a scaffold for soft tissue augmentation, specifically in the treatment of facial wrinkles and folds. The study followed a group of 20 patients who underwent facial injections with porous PLA and evaluated their skin morphology over a period of 5 years. The results showed that the porous PLA was well tolerated and maintained its shape and structure over the 5-year period. The authors also observed that the PLA scaffold was gradually replaced by native tissue, with no significant inflammation or adverse reactions. The study suggests that porous PLA is a promising material for soft tissue augmentation, with potential long-term benefits (Level 2b).

Abu Hajleh et al. [13] presented a mini-review of the various delivery systems of polylactic acid (PLA), a biodegradable polymer commonly used in cosmetic and pharmaceutical applications. The authors discussed the different forms of PLA, including nanoparticles, microspheres, and films, and their potential applications in cosmetics, such as skin care and hair care products. They also reviewed the advantages and limitations of each delivery system, including their stability, release kinetics, and compatibility with skin. The authors highlight the importance of understanding the physical and chemical properties of PLA in different formulations to optimize its performance and safety. They concluded that nano- and micro-particulate systems of PLA offer improved delivery and stability compared to traditional formulations, making them promising for future cosmetic applications (Level 5).

Oh et al. [14] investigated the effects of poly-D, L-lactic acid (PDLLA) on skin aging in animals. The researchers found that PDLLA treatment increased angiogenesis (the formation of new blood vessels) and collagen synthesis in aged animal skin, leading to improved skin elasticity and reduced wrinkle formation. The study used a mouse model of skin aging and found that PDLLA treatment increased the expression of genes involved in angiogenesis and collagen synthesis, as well as enhanced the activity of enzymes involved in collagen synthesis. The authors suggested that PDLLA may be a promising treatment for skin aging, as it promotes the formation of new blood vessels and improves collagen production, which can help to restore youthful skin appearance (Level 1c).

Gao et al. [15] compared the efficacy and safety of poly (L-lactic acid) microspheres (PLLA) as dermal fillers to other common fillers. The authors conducted a comprehensive review of existing studies on PLLA and other fillers, including hyaluronic acid, calcium hydroxylapatite, and polyacrylamide. The results showed that PLLA microspheres had superior biocompatibility, biodegradability, and durability compared to other fillers. PLLA microspheres were also found to be more effective in treating wrinkles and skin folds, and had fewer adverse reactions compared to other fillers. The authors suggested that PLLA microspheres may be a suitable alternative to traditional dermal fillers due to their superior properties and potential for long-term safety (Level 1a).

Guo et al. [16] provided a comprehensive review of injectable fillers, including their current status, physicochemical properties, function mechanism, and perspectives. The authors discussed the advantages and disadvantages of different types of fillers, including poly-D, L-lactic acid (PDLLA). They highlight that PDLLA has been widely used as a dermal filler due to its biocompatibility, biodegradability, and ability to stimulate collagen production. The authors also discussed the physicochemical properties of PDLLA, including its high viscosity, which allows it to maintain its shape and provide long-term support to the skin. Additionally, they mentioned that PDLLA has been shown to be effective in treating various types of wrinkles and skin folds, and has few adverse reactions (Level 1c).

Ren et al. [17] discussed the application of biodegradable poly (L-lactic acid) (PLLA) in biomedical materials. The authors provided an overview of the synthesis, modification, processing, and applications of PLLA. They described the synthesis methods of PLLA, including ring-opening polymerization and condensation polymerization, and its modification through copolymerization and blending with other biodegradable polymers. The authors also discussed the processing methods of PLLA, including extrusion, injection molding, and solvent casting, and its applications in biomedical materials, such as sutures, implants, and tissue engineering scaffolds. They highlighted the advantages of PLLA, including its biocompatibility, biodegradability, and ability to stimulate collagen production (Level 3b).

**Figure 3:**
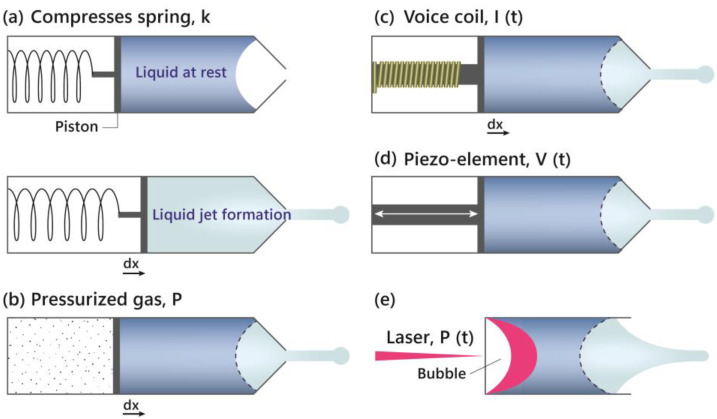
Needleless injectors utilizing various force mechanisms for liquid jet formation. (**a**) Compressed spring: a spring is compressed, and when released, it drives a piston that pushes the liquid to form a jet. (**b**) Pressurized gas: a chamber filled with pressurized gas expels the liquid by driving a piston forward, creating a jet. (**c**) Voice coil: an electromagnetic voice coil generates motion when current is applied, moving the piston and creating a liquid jet. (**d**) Piezo-element: a piezoelectric element expands and contracts under an applied voltage, propelling the liquid to form a jet. (**e**) Laser-induced: a laser creates a bubble within the liquid, generating pressure that drives the liquid out as a jet. Each modality demonstrates a different approach to achieve high-speed liquid injection without the use of needles.

Seo et al. [9] highlighted the efficacy of using laser-induced micro-jet injectors with PDLLA (Juvelook, VAIM Inc., Republic of Korea) for the treatment of striae distensae (SD). This method leverages the properties of PDLLA, which include biodegradability and biocompatibility, to stimulate collagen synthesis, thereby promoting skin regeneration and improving the appearance of stretch marks (Figure 3). In a study involving four female participants, significant improvements were observed after 5–7 treatment sessions, with minimal side effects such as petechiae. This innovative approach, which utilizes high-speed jet pressure to deliver PDLLA into the skin, has shown promising results in enhancing skin texture, elasticity, and overall appearance, offering a minimally invasive and effective alternative to traditional SD treatments (Level 5).

PDLLA is highly effective in aesthetic applications due to its biodegradability, biocompatibility, and ability to stimulate collagen synthesis, making it ideal for skin rejuvenation, scar remodeling, and wound healing, though potential complications such as granulomas, nodules, and filler migration require careful management and practitioner training.

## 3. PDLLA Complication and Management

While Poly-D,L-lactic acid (PDLLA) has proven to be highly effective in various aesthetic applications, its use is not without potential complications. One of the primary concerns with PDLLA injections is the formation of granulomas, which are firm, erythematous nodules that can arise at the injection site due to allergic reactions, foreign body responses, or delayed hypersensitivity. Other complications include the development of non-inflammatory nodules, often resulting from uneven distribution of the biostimulator during the injection process. Additionally, filler migration can occur, leading to asymmetry, swelling, and, in severe cases, multifocal strokes and vision loss if the filler inadvertently enters the bloodstream. Effective management of these complications involves early diagnosis and treatment, such as the use of topical or intralesional corticosteroids for granulomas, and ensuring even distribution techniques to prevent nodule formation. Thorough patient assessment and informed consent are critical to mitigate risks, and ongoing practitioner training is essential to maintain high standards of care. As the understanding of PDLLA’s mechanisms and potential side effects improves, strategies for minimizing and managing complications continue to evolve, enhancing the safety and efficacy of PDLLA-based treatments (Table 2).

Perez Willis et al. [18] reported a case study of a patient who developed a granuloma after receiving injections of poly-D, L-lactic acid (PDLLA) for skin rejuvenation. The patient presented with a firm, erythematous, and tender nodule at the injection site, which was diagnosed as a granuloma. The patient was treated with topical triamcinolone, which led to a significant improvement in the lesion. The authors discussed the potential mechanisms of granuloma formation after PDLLA injection, including an allergic reaction, foreign body reaction, or delayed hypersensitivity. The study highlights the importance of monitoring patients for adverse reactions after PDLLA injection and the need for early treatment of any suspected complications (Level 5).

Lin et al. [19] presented a study on the importance of evenly distributing biostimulators in the skin to prevent non-inflammatory nodules from forming. The authors investigated the effects of uneven distribution of biostimulators on the formation of nodules, which are a common complication of cosmetic injections. The study found that when biostimulators are not evenly distributed, they can accumulate and cause inflammation, leading to the formation of nodules. The authors also discovered that the uneven distribution of biostimulators can be due to various factors, including the injection technique used by the practitioner, the type of biostimulator used, and the individual’s skin characteristics. The study suggests that practitioners should take steps to ensure even distribution of biostimulators during injections to prevent non-inflammatory nodules from forming (Level 2b).

Wollina et al. [20] presented a narrative review of filler migration after facial injection. The authors discussed the common causes of filler migration, including incorrect injection technique, inadequate placement of the filler, and individual patient factors such as skin type and anatomy. They also reviewed the various types of fillers and their potential for migration, including hyaluronic acid, calcium hydroxylapatite, and poly-L-lactic acid. The authors highlighted the importance of proper patient selection and informed consent, as well as the need for adequate training and experience in performing facial injections. They also discussed the potential consequences of filler migration, including asymmetry, swelling, and inflammatory reactions. The authors concluded that filler migration is a common complication of facial injection and that a thorough understanding of its causes and consequences is essential for optimal patient care (Level 5).

Tan et al. [21] reported on a case series of patients who experienced multifocal strokes and vision loss following polydioxanone (PDLLA) filler injections. The authors presented a retrospective review of 10 patients who received PDLLA filler injections for facial rejuvenation and cosmetic purposes. The patients experienced sudden onset of vision loss, often accompanied by headache, nausea, and vomiting. Imaging studies revealed multifocal strokes in the posterior cerebral artery territory, which were thought to be related to the filler injections. The authors suggested that the strokes may have been caused by the injection procedure, which led to the introduction of air bubbles into the blood stream. The authors also highlighted the importance of careful patient selection and informed consent before undergoing PDLLA filler injections (Leve 2c).

Wang et al. [22] reported a case of a 35-year-old woman who experienced multiple branch retinal artery occlusions (BRACOs) following the injection of a new facial cosmetic filler, Poly-D, L-lactic Acid (PDLLA). The patient received injections for facial rejuvenation and reported sudden onset of blurred vision and loss of vision in her left eye. Fundus examination revealed BRACOs in multiple branches of the retinal artery. The authors suggested that the BRACOs may have been caused by the injection procedure, which led to the introduction of air bubbles into the retinal artery. The patient was treated with antiplatelet therapy and had a good recovery. The authors highlighted the importance of careful patient selection, informed consent, and proper training for physicians administering facial cosmetic fillers (Level 4).

PDLLA injections are effective for aesthetic applications but can lead to complications such as granulomas, non-inflammatory nodules, and filler migration, necessitating careful management through early diagnosis, appropriate treatment, and practitioner training.

## 4. Management of Atrophic Acne Scar

The management of atrophic acne scars has been significantly advanced by the use of needle-free jet injection of poly-(Lactic Acid) (PLA), which stimulates collagen production and improves skin texture with minimal invasiveness and high patient satisfaction.

Rho et al. [23] presented a review of the literature on needle-free jet injection (NFI) of poly-(Lactic Acid) (PLA) for the treatment of atrophic acne scars. The authors discussed the current state of knowledge on NFI, including its mechanisms, advantages, and limitations. They also provided a detailed review of the available literature on the use of NFI-PLA for the treatment of atrophic acne scars, including its efficacy, safety, and patient satisfaction. The authors also presented two clinical cases that demonstrate the effectiveness of NFI-PLA in treating atrophic acne scars. The study highlights the potential benefits of NFI-PLA, including its minimally invasive nature, reduced pain and discomfort, and improved patient satisfaction (Level 5).

The use of needle-free jet injection of PLA for atrophic acne scars has advanced treatment by stimulating collagen production, improving skin texture, and achieving high patient satisfaction with minimal invasiveness.

## 5. Composition and Combination of PDLLA with Other Treatment Modalities

The composition and combination treatments of Poly-D,L-lactic acid (PDLLA) play a pivotal role in enhancing its efficacy for aesthetic and medical applications. PDLLA’s unique chemical structure, characterized by a higher molecular weight and uniform monomer composition compared to other lactic acid polymers, provides superior stability and biocompatibility. These properties make PDLLA an excellent candidate for soft tissue augmentation and skin rejuvenation. Additionally, PDLLA can be effectively combined with other treatments to amplify its benefits. For instance, the combination of PDLLA with platelet-rich plasma (PRP) has shown remarkable results in treating deep nasolabial folds, significantly improving skin elasticity, firmness, and texture. Furthermore, innovative delivery methods, such as laser-generated needle-free microjet injectors, enhance the precision and effectiveness of PDLLA administration, making the treatment minimally invasive and well tolerated. These advancements highlight the versatility and potential of PDLLA in developing comprehensive and synergistic treatment protocols that address a wide range of dermatological and aesthetic concerns (Table 3).

Chen et al. [24] compared the compositions of injectable poly-D, L-lactic acid (PDLLA) and injectable poly-L-lactic acid (PLLA) in terms of their chemical structures and physical properties. The authors analyzed the molecular weights, monomer compositions, and thermal degradation temperatures of both materials. The results show that PDLLA has a higher molecular weight and a more uniform monomer composition compared to PLLA. The glass transition temperature, melting temperature and tensile strength of PDLLA are all lower than those of PLLA, and the degradation time of PDLLA is faster than that of PLLA. The study also investigated the effects of the materials on human skin fibroblasts, finding that both PDLLA and PLLA are biocompatible and non-toxic (Level 3b).

Pratama et al. [25] presented a case report on the combination of platelet-rich plasma (PRP) and poly-D, L-lactic acid (PDLLA) for the treatment of deep nasolabial folds. The authors treated a 45-year-old woman with significant nasolabial folds using a combination of PRP and PDLLA injections. The treatment was administered at 2-month intervals over a period of 6 months. The results showed significant improvements in the depth and appearance of the nasolabial folds, with a notable increase in skin elasticity and firmness. The patient also reported improved facial texture and reduced fine lines and wrinkles. The authors concluded that the combination of PRP and PDLLA may be a promising treatment for improving deep nasolabial folds, offering a minimally invasive and relatively painless alternative to surgical procedures (Level 5).

Oh et al. [26] presented a study on the use of a laser-generated needle-free microjet injector to deliver poly-D, L-lactic acid (PDLLA) for facial skin rejuvenation. The authors treated 20 patients with mild-to-moderate facial wrinkles and skin laxity using the microjet injector. The treatment was administered in a single session, with PDLLA injections made in a grid pattern on the face. The results showed significant improvements in facial wrinkles, skin texture, and elasticity, as well as a reduction in skin fold thickness and improvement in skin brightness. The study also evaluated the safety and tolerability of the treatment, finding that it was well tolerated and no serious adverse effects were reported. The authors concluded that the use of a laser-generated needle-free microjet injector for PDLLA delivery may be a promising treatment for facial skin rejuvenation, offering a minimally invasive and relatively painless alternative to traditional injectable methods (Level 4).

The studies highlighted the potential of PDLLA in enhancing dermatological treatments through its stable and biocompatible properties, innovative delivery methods, and effective combination therapies, demonstrating significant improvements in skin conditions and warranting further research.

## 6. Dermatology Condition and Wound-Healing Application

Recent studies have demonstrated the potential of nanotechnology and advanced materials in enhancing dermatological treatments and wound healing, showcasing innovations such as surfactin-stabilized nanoparticles, PLA nanoparticles for drug delivery, airbrushed nanofibers with bioactive cores, electrospun fibers with natural compounds, nanotechnology-based combination drug therapy for skin cancer, and poly-D, L-lactic acid microspheres as biocompatible subdermal fillers (Table 4).

Lewińska et al. [27] presented a study on the development of surfactin-stabilized PDLLA nanoparticles for potential skin application. The authors used a combination of surfactin, a biosurfactant, and PDLLA to create nanoparticles that could be used for skin delivery of bioactive molecules. The nanoparticles were characterized in terms of their size, shape, and stability, and were found to be stable in aqueous solution and resistant to enzymatic degradation. The authors also evaluated the nanoparticles’ ability to encapsulate and release model bioactive molecules, such as fluorescein isothiocyanate (FITC), and found that the nanoparticles were able to efficiently release the molecules over a period of several days. The study suggested that surfactin-stabilized PDLLA nanoparticles could be a promising vehicle for skin delivery of bioactive molecules, offering advantages such as improved stability, controlled release, and enhanced bioavailability (Level 3a).

Rancan et al. [28] presented a study on the use of polylactic acid (PLA) nanoparticles as drug delivery systems for local dermatotherapy. The authors synthesized PLA nanoparticles and evaluated their potential for delivering a model drug, the anti-inflammatory agent, dexamethasone, to the skin. The nanoparticles were found to have a size range of 50–100 nm, and their surface was modified with a hydrophilic polymer to enhance their ability to interact with skin cells. The authors evaluated the nanoparticles’ ability to deliver dexamethasone to the skin and found that they were able to effectively target the skin and deliver the drug over a period of several days. The study also evaluated the biocompatibility and biodegradability of the nanoparticles, finding that they were well tolerated and fully degraded in the skin (Level 3a).

Papakostas et al. [29] provided a comprehensive review of the current state of nanoparticles in dermatology. The authors discussed the potential applications of nanoparticles in various dermatological fields, including skin targeting, transdermal delivery, and skin imaging. They also highlighted the challenges and limitations associated with nanoparticle-based therapies, such as skin penetration, toxicity, and immunogenicity. The authors reviewed the different types of nanoparticles that have been investigated for dermatological applications, including liposomes, polymeric nanoparticles, and gold nanoparticles. They also discussed the use of nanoparticles in the treatment of skin diseases such as psoriasis and atopic dermatitis. The review concluded by highlighting the need for further research to fully understand the potential benefits and risks of nanoparticle-based therapies in dermatology (Level 1a)

Singh et al. [30] presented a novel approach to wound healing using airbrushed nanofibers with a bioactive core and antibacterial shell. The authors designed and synthesized nanofibers containing a bioactive molecule, such as platelet-derived growth factor (PDGF), as the core and an antibacterial agent, such as ciprofloxacin, as the shell. The nanofibers were fabricated using an airbrush technique and characterized for their morphology, size, and bioactivity. The authors demonstrated that the nanofibers effectively promoted wound healing by enhancing cellular adhesion, migration, and proliferation, while also inhibiting bacterial growth. The antibacterial properties of the nanofibers reduced bacterial colonization and inflammation, which can impede wound healing. The authors concluded that the airbrushed nanofibers with a bioactive core and antibacterial shell have potential for wound-healing applications (Level 2a).

Hermosilla et al. [31] presented a review of the use of electrospun fibers loaded with natural bioactive compounds as a biomedical system for skin burn treatment. The authors discussed the importance of effective wound-healing strategies, particularly for skin burns, which can be challenging to treat due to their complex nature and potential for infection. The authors highlighted the potential benefits of using electrospun fibers, which can be designed to mimic the natural structure of skin, as well as loaded with natural bioactive compounds such as antioxidants, antimicrobials, and growth factors. The review discussed the various natural bioactive compounds that have been used in electrospun fibers, including flavonoids, polyphenols, and saponins, and their potential to enhance wound healing by promoting tissue regeneration, reducing inflammation, and inhibiting bacterial growth. The authors concluded that electrospun fibers loaded with natural bioactive compounds have shown promise in preclinical studies and may be a promising approach for skin burn treatment (Level 5).

Kumari et al. [32] provided a comprehensive review of the recent advances in nanotechnology-based combination drug therapy for skin cancer. The authors highlighted the limitations of traditional chemotherapy and radiation therapy for skin cancer, which often lead to toxic side effects and resistance to treatment. They discussed the potential benefits of using nanotechnology-based combination drug therapy, which can improve the delivery of drugs to cancer cells, enhance their efficacy, and reduce side effects. The authors reviewed the various types of nanocarriers, including liposomes, nanoparticles, and micelles, that have been used to deliver combination drug therapy to skin cancer. They also discussed the different types of drugs that have been used in combination therapy, including chemotherapy agents, immunomodulators, and hormonal agents. The authors concluded that nanotechnology-based combination drug therapy has shown promise in preclinical studies and may be a promising approach for the treatment of skin cancer (Level 5).

Wang et al. [33] reviewed the recent advances in nanotechnology-based transdermal drug delivery for targeted tumor therapy. The authors highlighted the challenges of traditional chemotherapy, including low bioavailability, poor targeting, and severe side effects. They discussed the potential of nanotechnology-based transdermal drug delivery, which can enhance the bioavailability of drugs, improve targeting to cancer cells, and reduce side effects. The authors reviewed the various types of nanoparticles, including liposomes, polymeric nanoparticles, and lipid-based nanoparticles, that have been used for transdermal delivery of anticancer drugs. They also discussed the different methods of transdermal delivery, including iontophoresis, sonophoresis, and electroporation. The authors concluded that nanotechnology-based transdermal drug delivery has shown promise in preclinical studies and may be a promising approach for targeted tumor therapy (Level 5).

Lin et al. [34] presented a preclinical study to evaluate the efficacy and safety of poly-D, L-lactic acid microspheres (PDLLA) as subdermal fillers in animals. The authors injected PDLLA microspheres into the backs of mice and rabbits, and then evaluated the resulting tissue responses at different time points. The results showed that PDLLA microspheres were well tolerated and did not cause significant inflammation or tissue damage. The microspheres were also found to be slowly degraded and absorbed over time, with a peak effect at 12 weeks. The authors suggested that PDLLA microspheres may be a suitable alternative to traditional dermal fillers due to their biocompatibility and biodegradability (Level 1c).

Recent studies highlight the potential of polylactic acid (PDLLA) and nanotechnology-based materials in improving dermatological treatments and wound healing through enhanced stability, controlled release, and targeted therapies, necessitating further research for comprehensive validation.

## 7. Discussion

PDLLA is highly effective in aesthetic applications due to its biodegradability, biocompatibility, and collagen-stimulating properties, making it ideal for skin rejuvenation, scar remodeling, and wound healing. However, its use can lead to complications such as granulomas, non-inflammatory nodules, and filler migration, requiring careful management through early diagnosis and appropriate treatment. Needle-free jet injection of PDLLA has advanced the management of atrophic acne scars by stimulating collagen production and improving skin texture with minimal invasiveness. The combination treatments of PDLLA, such as with platelet-rich plasma (PRP) or innovative delivery methods like laser-generated microjet injectors, further enhance its efficacy in aesthetic and medical applications. Recent studies highlight the potential of PDLLA and nanotechnology-based materials in improving dermatological treatments and wound healing through enhanced stability, controlled release, and targeted therapies, warranting further research for comprehensive validation.

PDLLA demonstrates superior mechanical stability and biocompatibility compared to poly(D-lactic acid) (PDLA) and poly(L-lactic acid) (PLLA), primarily due to its unique stereoisomeric composition, which results in an amorphous polymer structure. This amorphous nature confers it several advantages. Firstly, it prevents the formation of large crystalline regions typical of PDLA and PLLA. Crystalline regions can render polymers brittle and prone to cracking under stress. In contrast, the lack of crystallinity in PDLLA results in a more uniform and flexible material, enhancing its mechanical stability and reducing susceptibility to mechanical failure.

Secondly, PDLLA degrades in a more controlled and predictable manner compared to the more crystalline PDLA and PLLA. The homogeneous distribution of D- and L-monomers in PDLLA allows for a uniform hydrolytic degradation process. This controlled degradation is crucial in biomedical applications where the timing of polymer breakdown affects the release of therapeutic agents and the healing process.

Thirdly, the amorphous nature of PDLLA also contributes to its better biocompatibility. Crystalline polymers can elicit more significant inflammatory responses due to the presence of sharp crystalline fragments during degradation. In contrast, a more gradual and uniform degradation of PDLLA produces smoother degradation products, less likely to provoke an adverse immune response. Additionally, PDLLA’s degradation products, primarily lactic acid, are naturally metabolized by the body, further enhancing its biocompatibility.

**Figure 4:**
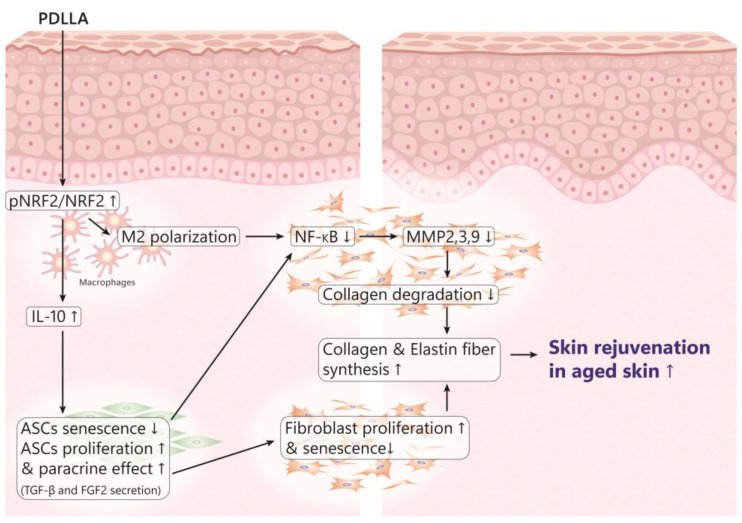
This figure illustrates the biochemical pathways and cellular interactions through which PDLLA contributes to skin rejuvenation in aged skin. PDLLA initiates a series of responses starting with the phosphorylation and activation of nuclear factor erythroid 2-related factor 2 (NRF2). This activation promotes the polarization of macrophages towards the anti-inflammatory M2 phenotype, which increases the production of interleukin-10 (IL-10). The elevated IL-10 levels lead to reduced senescence and increased proliferation of adipose-derived stem cells (ASCs), enhancing their paracrine effects through the secretion of transforming growth factor-beta (TGF-β) and fibroblast growth factor 2 (FGF2). Arrows pointing up represents upregulation while downward represents down regulation.

PDLLA exhibits several key mechanisms of action that contribute to its effectiveness in dermatological applications, with a significant emphasis on its anti-inflammatory properties. These mechanisms are intricately linked to the modulation of macrophages and adipose-derived stem cells (ASCs) in aged skin, as detailed in recent studies. One of the primary ways PDLLA exerts its anti-inflammatory effects is through the modulation of macrophages, specifically by increasing polarization towards the M2 phenotype. Macrophages can exist in different states, with M1 macrophages typically associated with pro-inflammatory responses and M2 macrophages linked to anti-inflammatory effects. PDLLA enhances the expression of nuclear factor (erythroid-derived 2)-like-2 factor (NRF2), a critical transcription factor that helps mitigate oxidative stress and inflammation (Figure 4).

Studies have shown that PDLLA treatment increases the phosphorylation of NRF2 in senescent macrophages, promoting M2 polarization. This shift towards the M2 phenotype is accompanied by elevated levels of interleukin-10 (IL-10), an anti-inflammatory cytokine known for its role in promoting cell survival and reducing inflammation. This process helps counteract the inflammatory environment typically seen in aged or damaged skin.

PDLLA also positively influences ASCs, which play a crucial role in skin regeneration and repair. Conditioned media from macrophages treated with PDLLA showed reduced markers of senescence and increased proliferation in ASCs. This improvement in ASC function is likely due to the increased IL-10 levels from the modulated macrophages. IL-10 enhances the survival and paracrine functions of ASCs, which, in turn, secrete beneficial factors such as transforming growth factor-β (TGF-β) and fibroblast growth factor 2 (FGF2). These factors are vital for promoting collagen synthesis and improving the extracellular matrix (ECM).

Another significant aspect of PDLLA’s anti-inflammatory action is its effect on reducing the expression of nuclear factor kappa-light-chain-enhancer of activated B cells (NF-κB) and matrix metalloproteinases (MMPs) in fibroblasts. NF-κB is a key regulator of inflammation and is often upregulated in aged or damaged skin, leading to increased MMP activity, which degrades collagen and other ECM components. PDLLA treatment, through its action on macrophages and ASCs, reduces NF-κB activation and MMP expression, thereby mitigating the breakdown of the ECM and promoting a more favorable environment for skin repair and rejuvenation.

In vivo studies on aged animal skin further support these findings, showing that PDLLA injections result in increased expression of NRF2, IL-10, and collagen types 1a1 and 3a1, alongside enhanced ASC proliferation. These changes collectively contribute to improved skin structure and reduced signs of aging.

PDLLA’s mechanisms of action in dermatology can be attributed to its unique properties. One of the most important mechanisms is its ability to stimulate collagen synthesis. PDLLA has been shown to increase collagen production by activating fibroblasts, the primary cells responsible for producing collagen. This increased collagen production leads to improved skin elasticity, firmness, and hydration [27].

Another mechanism of action is its ability to promote tissue regeneration. PDLLA’s biodegradable and biocompatible nature allows it to be easily absorbed by the body, stimulating the body’s natural healing processes. This promotes the growth of new tissue, which can lead to improved wound healing and scar remodeling [35].

PDLLA has also been shown to exhibit anti-inflammatory properties, which can contribute to its mechanisms of action in dermatology. Inflammation is a common underlying factor in many skin-related disorders, including acne, psoriasis, and atopic dermatitis. By reducing inflammation, PDLLA may help to alleviate symptoms and improve outcomes in these conditions [29].

PDLLA has been investigated for its potential applications in various clinical settings in dermatology. One of the most promising areas is wound healing. PDLLA has been shown to improve wound contraction rates, increase collagen production, and enhance tissue elasticity, making it a promising material for wound care [30].

PDLLA has also been explored for its potential use in skin rejuvenation. Its ability to stimulate collagen synthesis and promote tissue regeneration makes it an attractive material for improving skin elasticity and reducing fine lines and wrinkles [8,10,26]. In addition, PDLLA has been investigated for its potential use in scar remodeling. Its ability to stimulate collagen synthesis and promote tissue regeneration makes it a promising material for improving scar appearance and reducing the risk of hypertrophic scarring [23].

The efficacy and safety of PDLLA have been investigated in various clinical trials. The results have shown that PDLLA is generally well tolerated and safe for use in dermatological applications. In terms of efficacy, PDLLA has been shown to improve wound-healing outcomes, reduce fine lines and wrinkles, and improve scar appearance.

Despite the promising results of PDLLA in dermatology, several limitations and future directions need to be addressed. One main limitation is the lack of standardization in PDLLA preparation and administration methods, which can make it difficult to compare results across different studies. Another limitation is the need for further research on the optimal dosage and duration of PDLLA treatment for different clinical applications. Additionally, more research is needed to fully understand the mechanisms of action of PDLLA and how it interacts with different skin types and conditions.

Future directions for PDLLA research include investigating its potential use in combination with other biomaterials or treatments. For example, combining PDLLA with platelet-rich plasma (PRP) may enhance its efficacy in wound healing and skin rejuvenation [25].

While PDLLA has been extensively explored for its applications in dermatology, its potential extends far beyond this field. The versatility of PDLLA makes it an attractive material for a wide range of medical applications. One area where PDLLA has shown promise is in regenerative medicine. The ability of PDLLA to degrade slowly and release therapeutic molecules over time makes it an ideal material for tissue engineering applications [36,37,38,39].

PDLLA has also been explored for its use in the delivery of proteins and peptides. Its ability to degrade slowly and release therapeutic molecules over time makes it an ideal material for protein delivery applications [40,41,42,43]. Furthermore, PDLLA has been explored for its use in developing implantable devices. The biocompatibility and biodegradability of PDLLA make it an attractive material for developing implantable devices such as pacemakers and neurostimulators [44,45,46,47,48].

Additionally, PDLLA has been investigated for its use in treating orthopedic disorders. Its ability to degrade slowly and release therapeutic molecules over time makes it an ideal material for developing scaffolds for bone tissue engineering [38,49,50,51].

## 8. Conclusions

In conclusion, this literature review on the application of PDLLA in dermatology underscores its significant potential and current limitations within various clinical contexts. PDLLA’s unique properties, such as its ability to stimulate collagen synthesis, promote tissue regeneration, and exhibit anti-inflammatory effects, render it an appealing biomaterial for enhancing skin elasticity, firmness, and hydration. Evidence indicates that PDLLA has yielded promising outcomes in wound healing, skin rejuvenation, and scar remodeling. Its efficacy in improving wound contraction rates, boosting collagen production, and enhancing tissue elasticity positions it as a valuable material for wound care. Furthermore, its capacity to reduce fine lines and wrinkles, along with its beneficial impact on scar appearance, highlights its potential in skin rejuvenation and scar remodeling.

Despite these promising findings, several limitations and future research directions need to be addressed. There is a need for comprehensive studies to fully elucidate PDLLA’s mechanisms of action and its interactions with various skin types and conditions. Additionally, optimizing the dosage and duration of PDLLA treatments for different clinical applications requires further investigation. While these limitations present challenges, PDLLA’s biodegradable and biocompatible nature offers significant advantages for minimally invasive procedures, potentially reducing patient discomfort and improving clinical outcomes.

To fully realize PDLLA’s potential in dermatology, further research is essential. This includes extensive clinical trials to confirm its efficacy and safety across different clinical settings. Advancing our understanding of PDLLA’s mechanisms of action and optimal usage could pave the way for the development of innovative treatments and therapies, ultimately enhancing patient outcomes and quality of life.

## Figures and Tables

**Table 1:** This table summarizes various studies that explore the use of Poly-D,L-lactic acid (PDLLA) in aesthetic applications. It includes information on the focus of each study, the number of participants, the method used, the results obtained, and the level of evidence. The levels of evidence range from 1 (highest) to 5 (lowest), providing insight into the reliability and validity of each study.

Study	Focus	Participants	Method	Results	Level of Evidence
Seo et al. [5]	Skin rejuvenation with PDLLA (Juvelook) and NHA	20	Combination applied to facial skin	Improved skin elasticity, firmness, hydration, reduced wrinkles and fine lines	4
Ma et al. [8]	Rejuvenating dorsal hand skin	10	PDLLA injections at 3-month intervals	Improved skin texture, elasticity, wrinkle depth, skin brightness, no serious adverse effects	2b
Seo et al. [9]	Treating striae distensae with laser and PDLLA (Juvelook)	20	Ablative fractional CO2 laser + PDLLA (Juvelook) injections	Improved skin texture, elasticity, appearance of stretch marks, high patient satisfaction	2b
Lin et al. [10]	Nonsurgical lower eyelid rejuvenation	10	PDLLA injections at 3-month intervals	Improved skin texture, elasticity, wrinkle depth, skin brightness, no serious adverse effects	3b
Li et al. [11]	Soft tissue augmentation with PLLA/PLGA composites	-	Encapsulation in collagen matrix	High tensile strength, biocompatibility, supported cell growth and differentiation	3a
Ken et al. [12]	Long-term evaluation of porous PLA for facial wrinkles	20	Facial injections with porous PLA	Well tolerated, maintained shape over 5 years, replaced by native tissue	2b
Abu Hajleh et al. [13]	Review of PLA delivery systems	-	Various forms of PLA	Improved delivery and stability of nano-and micro-particulate systems	5
Oh et al. [14]	PDLLA effects on skin aging in animals	-	Animal model study	Increased angiogenesis, collagen synthesis, improved skin elasticity, reduced wrinkles	1c
Gao et al. [15]	Comparison of PLLA microspheres with other fillers	-	Review of existing studies	Superior biocompatibility, biodegradability, effectiveness, fewer adverse reactions	1a
Guo et al. [16]	Review of injectable fillers	-	Literature review	Emphasis on PDLLA’s biocompatibility, biodegradability, collagen stimulation	1c
Ren et al. [17]	Application of PLLA in biomedical materials	-	Review of synthesis and applications	Biocompatibility, biodegradability, collagen production stimulation	3b
Seo et al. [9]	Application of PDLLA (Juvelook) in combination with needless injection		Case study	Delivery of PDLLA	5

The numerical grading from 1 to 5 reflects the type of study design and methodological rigor, with Level 1 indicating systematic reviews or high-quality RCTs, Level 2 indicating a single high-quality RCT or strong quasi-experimental studies, Level 3 indicating cohort or case–control studies with less rigor, Level 4 indicating case series and poor-quality studies, and Level 5 indicating expert opinion or evidence based on physiology, while the alphabetical grading from a to c assesses consistency, quality, and applicability, with Grade a indicating strong consistency across high-quality studies, Grade b indicating moderate evidence with some inconsistency, and Grade c indicating weak evidence with significant flaws.

**Table 2:** This table details studies that address the complications associated with PDLLA use and their management strategies. It includes the focus of each study, key findings, and the level of evidence. This table highlights the importance of monitoring, early diagnosis, and appropriate treatment to manage and mitigate adverse effects such as granulomas, nodules, and filler migration.

Study	Focus	Findings	Level of Evidence
Perez Willis et al. [18]	Granuloma formation after PDLLA injection	Case study of granuloma treated with topical triamcinolone, discussion of mechanisms	5
Lin et al. [19]	Prevention of non-inflammatory nodules	Importance of even biostimulator distribution to prevent nodules	2b
Wollina et al. [20]	Filler migration after facial injection	Causes and consequences of filler migration, importance of proper technique	5
Tan et al. [21]	Multifocal strokes and vision loss after PDLLA filler injections	Case series, importance of careful patient selection and informed consent	2c
Wang et al. [22]	Branch retinal artery occlusions after PDLLA injections	Case report, importance of proper training and patient safety	4

The numerical grading from 1 to 5 reflects the type of study design and methodological rigor, with Level 1 indicating systematic reviews or high-quality RCTs, Level 2 indicating a single high-quality RCT or strong quasi-experimental studies, Level 3 indicating cohort or case–control studies with less rigor, Level 4 indicating case series and poor-quality studies, and Level 5 indicating expert opinion or evidence based on physiology, while the alphabetical grading from a to c assesses consistency, quality, and applicability, with Grade a indicating strong consistency across high-quality studies, Grade b indicating moderate evidence with some inconsistency, and Grade c indicating weak evidence with significant flaws.

**Table 3:** This combined table presents studies on the management of atrophic acne scars using PDLLA and its composition and combination treatments. It includes the focus of each study, key findings, and the level of evidence. The table highlights innovative approaches like needle-free jet injection and combination therapies that enhance PDLLA’s effectiveness in treating acne scars and other cosmetic concerns.

Study	Focus	Findings	Level of Evidence
Chen et al. [24]	Comparison of PDLLA and PLLA compositions	Higher molecular weight, uniform monomer composition, greater stability for PDLLA	3b
Pratama et al. [25]	Combination of PRP and PDLLA for nasolabial folds	Significant improvements in fold depth, skin elasticity, firmness	5
Oh et al. [26]	Laser-generated microjet injector for PDLLA delivery	Improved facial wrinkles, skin texture, elasticity, well tolerated	4

The numerical grading from 1 to 5 reflects the type of study design and methodological rigor, with Level 1 indicating systematic reviews or high-quality RCTs, Level 2 indicating a single high-quality RCT or strong quasi-experimental studies, Level 3 indicating cohort or case–control studies with less rigor, Level 4 indicating case series and poor-quality studies, and Level 5 indicating expert opinion or evidence based on physiology, while the alphabetical grading from a to c assesses consistency, quality, and applicability, with Grade a indicating strong consistency across high-quality studies, Grade b indicating moderate evidence with some inconsistency, and Grade c indicating weak evidence with significant flaws.

**Table 4:** This table summarizes studies on the use of PDLLA in various dermatological conditions and wound-healing applications. It includes the focus of each study, key findings, and the level of evidence. The table emphasizes the potential of PDLLA in innovative drug delivery systems, nanoparticles, and nanofibers for improving skin conditions and enhancing wound-healing processes.

Study	Focus	Findings	Level of Evidence
Lewińska et al. [27]	Surfactin-stabilized PDLLA nanoparticles	Stable nanoparticles, controlled release, enhanced bioavailability	3a
Rancan et al. [28]	PLA nanoparticles for local dermatotherapy	Effective drug delivery, biocompatibility, biodegradability	3a
Papakostas et al. [29]	Review of nanoparticles in dermatology	Applications, challenges, and potential benefits of nanoparticles	1a
Singh et al. [30]	Airbrushed nanofibers for wound healing	Enhanced wound healing, antibacterial properties, improved cellular functions	2a
Hermosilla et al. [31]	Electrospun fibers for skin burn treatment	Benefits of natural bioactive compounds in wound healing	5
Kumari et al. [32]	Nanotechnology-based drug therapy for skin cancer	Improved drug delivery, reduced side effects, enhanced efficacy	5
Wang et al. [33]	Transdermal drug delivery for tumor therapy	Enhanced bioavailability, targeted delivery, reduced side effects	5
Lin et al. [34]	Preclinical study of PDLLA microspheres as fillers	Good biocompatibility, biodegradability, sustained tissue response	1c

The numerical grading from 1 to 5 reflects the type of study design and methodological rigor, with Level 1 indicating systematic reviews or high-quality RCTs, Level 2 indicating a single high-quality RCT or strong quasi-experimental studies, Level 3 indicating cohort or case–control studies with less rigor, Level 4 indicating case series and poor-quality studies, and Level 5 indicating expert opinion or evidence based on physiology, while the alphabetical grading from a to c assesses consistency, quality, and applicability, with Grade a indicating strong consistency across high-quality studies, Grade b indicating moderate evidence with some inconsistency, and Grade c indicating weak evidence with significant flaws.

## Data Availability

Data are available by contacting the corresponding author.
